# *PLIN4*-related myopathy: clinical, histological and imaging data in a large cohort of patients

**DOI:** 10.1007/s00415-023-11729-8

**Published:** 2023-05-05

**Authors:** Lorenzo Maggi, Sara Gibertini, Eliana Iannibelli, Annamaria Gallone, Silvia Bonanno, Daniele Cazzato, Simonetta Gerevini, Marco Moscatelli, Flavia Blasevich, Giorgia Riolo, Renato Mantegazza, Alessandra Ruggieri

**Affiliations:** 1 Neuroimmunology and Neuromuscular Disease Unit, Fondazione IRCCS Istituto Neurologico Carlo Besta, Milan, Italy; 2 Neurophysiology Unit, Fondazione IRCCS Istituto Neurologico Carlo Besta, Milan, Italy; 3 Unit of Neuroradiology, Papa Giovanni XXIII Hospital, Bergamo, Italy; 4 Neuroradiology, Fondazione IRCCS Istituto Neurologico Carlo Besta, Milan, Italy; 5 Department of Biomedical Sciences for Health, University of Milan, Milan, Italy

Dear Sirs,

In an Italian kindred composed of 19 individuals affected by a rare autophagic vacuolar myopathy linked to chromosome 19p13.3 [1] we formerly identified the genetic cause by means of a multi-omic approach [2]. Notably, with long-read sequencing we depicted a large expansion, unvaried among all patients, within the region of *PLIN4* gene encoding for the amphipatic helix of perilipin 4, a protein member of the perilipins’ family. Perilipins coat and regulate the metabolism of lipid droplets (LDs) [3, 4] and perilipin 4 is the most represented in the muscle tissue [5], although its function is yet to be fully clarified [6]. We showed that in the affected patients, perilipin 4 is accumulating in the subsarcolemmal region of the fibers and within the vacuoles and this positivity is solely present in our patients and not in other tested vacuolar myopathies such as those caused by mutations in *DNAJB6*, *VMA21*, *GAA*, *GNE* and *LAMP2* [2]. The accumulation of perilipin 4 within the fibers, triggers the activation of the aggrephagy pathway players p62/SQSTM1, NBR1 and WDFY3 [7–9], disrupting the organization of the fibers thus altering their contractile abilities. We here report the comprehensive clinical and muscle imaging characterization of 15 patients from this family, carrying the *PLIN4* gene expansion.

In this retrospective monocentric study, patients were considered as affected when showing muscle weakness at neurological examination and carrying the coding expansion in *PLIN4* gene. Hence, an asymptomatic 23-year-old man (son of patient IV:17) displaying the expansion was excluded, showing a normal neurological examination and normal muscle MRI imaging at pelvis, thigh and leg levels. Patients’ pedigree is shown in Fig. 1. Disease severity at last visit was assessed according to Walton and Gardner and Medwin modified for proximal myopathy (WGM) [10] and inclusion body myositis-functional rating scale (IBM-FRS) [11]. We included 15 affected patients presenting, at mean age of 46.1 ± 10.1 years (range 30–66), with lower and/or upper limb distal muscle weakness in 12/14 (85.7%) of them, mostly in lower limbs; only 2/14 (14.3%) patients reported scapular or pelvic weakness at onset, respectively. Patients’ clinical features are summarized in Table 1. Furthermore, patient V:13 reported left calf atrophy presenting at the age of 42 years, developing mild bilateral big toe extension muscle weakness only at the age of 44 years. In addition, 2 (13.3%) patients presented with minimal muscle weakness at neurological examination: patient V:14 showing mild (MRC 4/5) distal and neck flexor muscle weakness since the age of 32 years and patient IV:19 displaying minimal (MRC 4.5/5) weakness of left foot dorsiflexion, at the age of 53 years. Their muscle biopsies revealed the presence of rimmed vacuoles despite demonstrating no detectable muscle weakness.

**Fig. 1:**
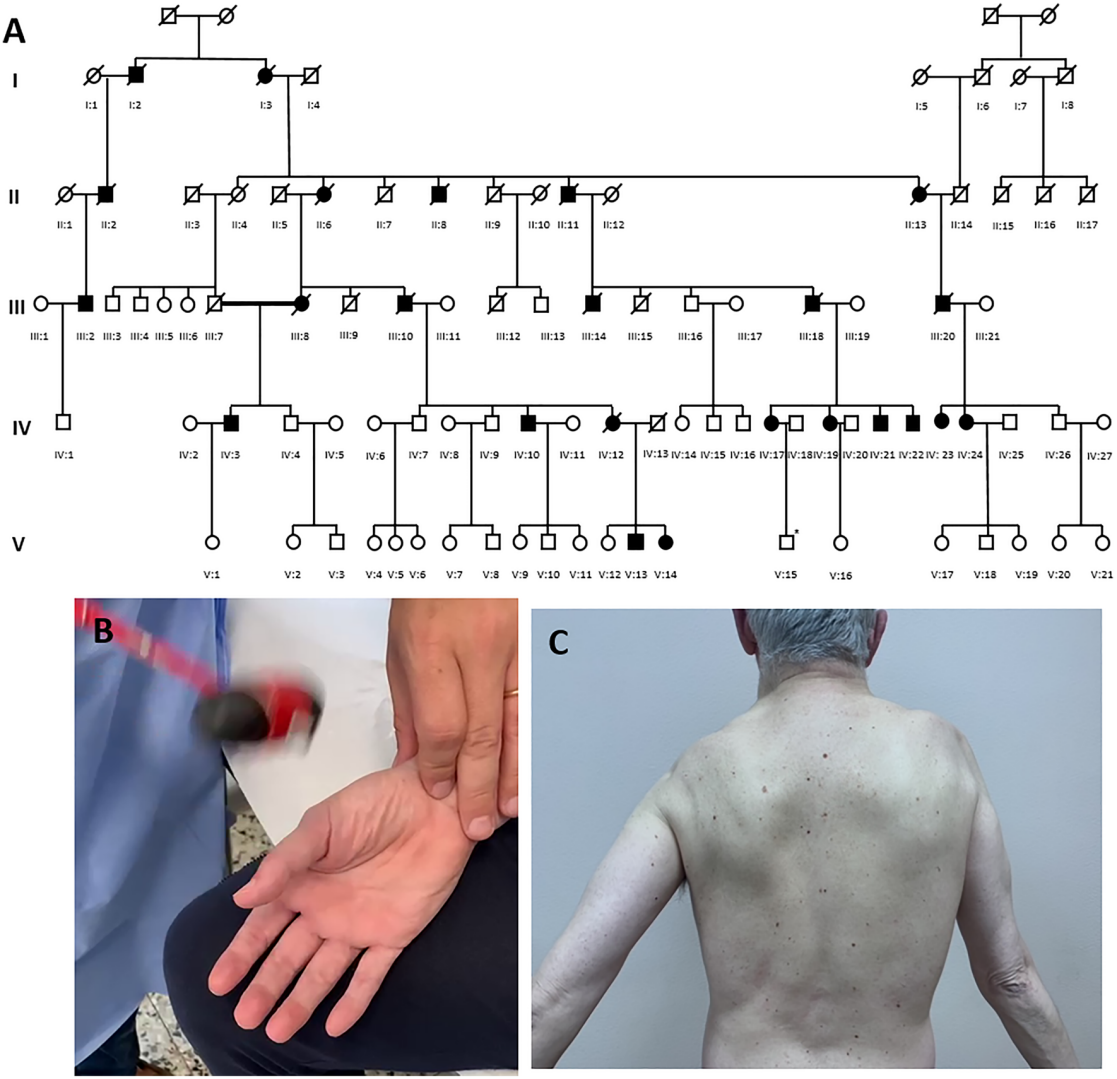
**A** Family pedigree. **B** Percussion myotonia at thenar eminence level. **C** Marked scapular muscle involvement with arm abduction weakness without scapular winging

**Table 1:** Patients’ clinical features

	IV:10	IV:12	III:8	IV:21	IV:22	IV:19	IV:17	III:18	III:14	IV:3	IV:24	IV:23	III:20	V:14	V:13
Gender/Mean age at onset (y)	M, 50	F, 42	F, 45	M, 30	M, 49	F, 53	F, 31	M, 66	M, 44	M, 54	F, 49	F, 46	M, 59	F, 32	M, 42
Presentation	Di UL	Di LL	Di UL + LL	Di LL	Pe	Di LL	Di UL	Di UL + LL	Di UL	Di LL	Sc	Di LL	Di LL	Di LL	Di LL*
Facial/ocular/bulbar involvement	No/no/no	No/no/yes	NA	No/no/no	No/no/no	No/no/no	No/no/no	NA	No/no/no	No/no/no	No/no/no	Yes/no/yes	No/no/no	No/no/no	No/no/no
Age at last visit (y)	66	69	73	44	56	54	52	71	58	72	55	55	65	38	44
Predominant weakness pattern at last visit	Generalized	Sc/Pe/Di LL	Di UL + LL	Sc/Pe/Di UL + LL	Sc/Pe/Di LL	Di LL	Di UL + LL	Sc/ Di UL + LL	Di UL + LL	Sc/Di UL + LL	Sc/Pe	Sc/Pe	Pe/Di LL	Ax/Di LL	Di LL
Contractures	Ankle	Ankle	Ankle	No	Ankle	No	No	No	Ankle	No	No	Ankle	Ankle	No	No
Wheelchaired (age, y)	Yes (63)	Yes (67)	Yes (85)	No	No	No	No	Yes (71)	Yes (58)	No	No	No	Yes (64)	No	No
Respiratory involvement	No	No	NA	Yes	No	No	No	NA	NA	No	No	No	Yes	No	No
CK range (IU)	NA	NA	NA	193	540–988	131	87–191	NA	460	NA	458	484	NA	275	545
Cardiac involvement	No	Yes (Ar)	No	No	No	No	No	NA	No		No	No	No	No	No
Myotonic discharges at EMG	Yes	NA	No	Yes	Yes	Yes	Yes	NA	NA	Yes	Yes	Yes	Yes	Yes	NA
Clinical myotonia	NA	NA	NA	No	Yes (P/S)	Yes (P)	Yes (P)	NA	NA	Yes (S)	No	Yes (P)	NA	No	No
Age at death (y)	No	69	94	No	No	No	No	72	65	No	No	No	72	No	No
WGM modified at last visit	8	8	NA	4	5	0	1	8	8	8	4	6	8	0	1
IBM-FRS at last visit	6	8	NA	32	25	40	33	NA	NA	17	33	27	NA	40	39
Rimmed vacuoles (age at biopsy -y-; muscle)	+ + (54; LQF)	+ + (47; QF)	NA	+ + (31; QF)	NA	− (54; QF)	+ (32; QF)	NA	NA	+ + (67; QF)	+ (43; QF)	+ + (53; TA)	NA	+ (34; QF)	+ (43; QF)
Positivity for PLIN4, FK2, p62, NBR1 on muscle	+ + + , + + + , + + + , + + +	+ + + , + + + , + + + , + + +	NA	+ + + , + + + , + + + , + + +	NA	+ , + , + , +	+ , + , + , +	NA	NA	+ + , + + , + + , + +	+ , + , + , + +	+ + + , + + + , + + , + +	NA	+ , + , + , NA,	+ + , + + , + + , + +

*y* years, *Pe* pelvic, *Di* distal, *Sc* scapular, *UL* upper limbs, *LL* lower limbs, *Ax* axial, *IU* international unit, *Ar* arrhythmia, *P* percussion, *S* spontaneous, *WGM* Walton and Gardner and Medwin modified for proximal myopathy, *IBM-FRS* inclusion body myositis functional rating scale, *QF* quadriceps femoris, *TA* tibialis anterior

*Only calf atrophy, without muscle weakness

Predominant pattern of muscle involvement at last visit still included lower limb distal muscles in 13/15 (86.7%) patients, although often combined to other defects, as distal upper limb, scapular and pelvic muscle weakness; only 2 (13.3%) patients showed predominant scapular and pelvic weakness without relevant distal involvement; interestingly, one of them presented with lower limb muscle weakness. Six (46.2%) and 2 (15.4%) out of 13 patients developed neck flexors and extensors weakness, respectively. Mild asymmetry of limb muscle involvement was observed in 6 out of 12 patients.

No ptosis or oculoparesis were reported. Mild facial weakness was reported only in patient IV:23. Dysphagia was reported only in patient IV:12 in end-stage disease. Mild tongue weakness was observed only in 2/13 (15.4%) patients. No rigid spine, scoliosis or scapular winging were observed in our cohort (Fig. 1). Tendon contractures were detected in 7/15 (46.7%) patients and always limited to the ankles and occurring in late-disease stages.

All patients who underwent electromyography (*n* = 9) showed diffuse myopathic findings and myotonic discharges. Notably, mild to moderate clinical myotonia was observed in 5 out of 9 (55.6%) patients, mostly as thenar eminence percussion myotonia, regardless of the presence of distal muscle weakness and usually not observed in younger patients; 2 (16.7%) patients displayed mild spontaneous handgrip myotonia with warm-up phenomenon and only 2 (16.7%) further patients (III:14 and IV:17) reported difficulty in relaxing handgrip, worsened by cold in the former. Creatin phosphokinase was normal or slightly increased (< 3 × upper normal value) in all patients, except for patient IV:22, whose CK values ranged from 728 to 988 UI. Only patient IV:12 showed cardiac involvement, characterized by late-onset atrial fibrillation without evidence of cardiomyopathy. Furthermore, no relevant central nervous system or nerve involvement was observed.

Last clinical evaluation was performed at a mean age of 58.3 ± 10.8 years (range 39–73).

No patients required assisted ventilation, except for III:20, who underwent tracheostomy at the age of 71 years. Among 8 patients with available forced vital capacity at last visit, only patient IV:21 showed abnormal values (47% and 32% in sitting and clinostatic position, respectively, expressed as percentage of the predicted value), with normal arterial gas blood analysis and normal oxygen saturation during night and the six-minute walking test. Data on MGW and IBM-FRS are shown in Table 1 and Fig. 1. Death occurred in 5 (33%) patients at a median age of 74.4 ± 11.3 years (range 65–94), all already wheelchaired, mainly for respiratory and cardiac complications.

A neuroradiologist evaluated each muscle using modified Mercuri score [12] on axial T1w muscle MRI or CT scan images passing through the middle section of thigh and calf. Scoring was based on the most affected of the two sides and the results are plotted in a heat map table. The heat map shows a progression of muscle fatty replacement with age. The first and most affected muscles are the medial head of gastrocnemius, tibialis anterior and semimembranosus, while the least affected are the rectus femoris, the short head of biceps femoris and tibialis posterior.

Figure 2 shows the heat map and selected images from the patients (3 patients from normal to moderate to severe, thigh and calf levels).

**Fig. 2:**
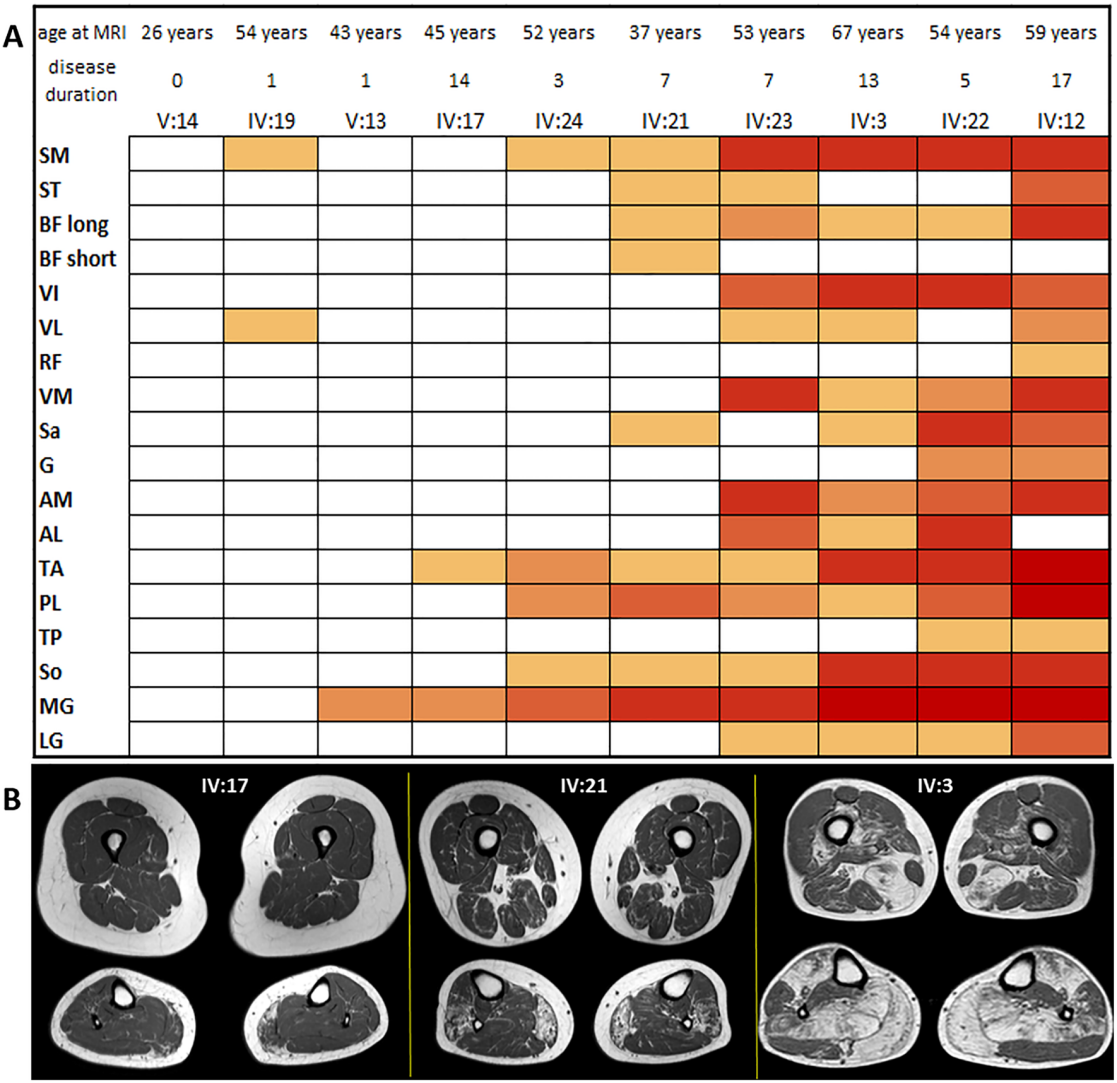
**A** Heat map. Patients and muscles are ordered according to the degree of fat replacement. We used the modified Mercuri score, with higher scores rendered with darker red. Age and years of symptoms at imaging are indicated **B** Thighs and calves section on Axial t1w images from three patients with increasing degree of fat replacement from left to right. *SM* semimembranosus, *ST* semitendinosus, *BF long* long head of the biceps femoris, *BF* *short* short head of the biceps femoris, *VI* vastus intermedius, *VL* vastus lateralis, *RF* rectus femoris, *VM* vastus medialis, *Sa* sartorius, *G* gracilis, *AM* adductor magnus, *AL* adductor longus, *TA* tibialis anterior, *PL* peroneus longus, *TP* tibialis posterior, *So* soleus, *MG* medial head of gastrocnemius, *LG* lateral head of gastrocnemius

We here describe clinical and imaging features of the largest cohort of *PLIN4*-related myopathy to date. This disease is characterized by adult presentation between the fourth and the seventh decade of life with distal lower and less frequently upper limb muscle weakness in most of the cases, suggesting that *PLIN4* gene expansion should be investigated in all patients presenting a distal myopathy, particularly in those showing rimmed vacuoles at muscle biopsy. Although remarkable distal muscle weakness was detected in 13/15 patients, relevant scapular and/or pelvic muscle weakness was observed in around 60% of the cases during the disease course. Disease progression is slow over the years, reaching its maximum severity within the beginning of the seventh decade of life, without any relevant cardiac or respiratory involvement, except for patient IV:21 developing severe restrictive lung disease during the fifth decade of life. However, 40% of the patients were wheelchaired-bound after a mean period of around 17 years from the onset. Recently, two studies, including 11 and 4 Chinese patients, respectively [13, 14] showed similar data, except for 2 familial cases with higher number of *PLIN4* repeat expansion presenting in third decade of life and displaying a more severe phenotype; moreover, these studies observed more frequent predominant proximal than distal muscle involvement at onset and during disease course. Notably, myotonic discharges were detected in all our patients who underwent electromyography, representing a further red flag for this disease entity. Percussion myotonia was observed in around half of the tested patients, but related symptoms were complained only by 2 patients, making clinical meaning of myotonia relatively poor.

Muscle MRI pattern did not show a complete correspondence with the other reports on *PLIN4*-related myopathy, particularly in the calves, where the soleus appeared to be more affected than the medial gastrocnemius; conversely, in our series, involvement of the latter always preceded the former. At thigh level, more similarities are observable among the studies, with relative sparing of gracilis and rectus femoris. This MRI pattern is relatively typical, presenting just some resemblance with dysferlinopathies [15].

Further studies are needed to clarify the molecular mechanisms of the disease and to improve the understanding of the genotype–phenotype correlation.

## Data Availability

The data reported in the current manuscript are available on reasonable request from the corresponding author.
